# 

**Published:** 1859-03

**Authors:** 


### MEDICAL AND SURGICAL SCIENCE.

EDITED BY AUSTIN FLINT, Jr., M. D.

Prof. of Physiology and Microscopic Anatomy in the Medical Department of the University of Buffalo,
One of the Surgeons to the Buffalo General Hospital.

BUFFALO, N. Y.:

A. I. MATHEWS, No. 220 MAIN STREET.

*LONDON:* H. BAILLIÈRE, 219 REGENT STREET. *PARIS:* J. B. BAILLIÈRE ET FILS, RUE HAUTEFEUILLE. *MADRID:* C. BAILLY-BAILLIÈRE, CALLE DEL PRINCIPE. *BERLIN & VIENNA:* AGENTS OF B. WESTERMANN & CO.

Price $2.50, payable in advance, or $3 00 if not paid till the end of the year.

